# FOXA1 Expression in Nasopharyngeal Carcinoma: Association with Clinicopathological Characteristics and EMT Markers

**DOI:** 10.1155/2020/4234632

**Published:** 2020-06-22

**Authors:** Nihel Ammous-Boukhris, Wajdi Ayadi, Mariem Derbel, Nesrine Allaya-Jaafar, Slim Charfi, Jamel Daoud, Tahya Sellami-Boudawara, Raja Mokdad-Gargouri

**Affiliations:** ^1^Center of Biotechnology of Sfax, University of Sfax, Sidi Mansour Street Km 6, BP 1177, 3038 Sfax, Tunisia; ^2^Department of Anatomo-pathology, Habib Bourguiba Hospital, Sfax, Tunisia; ^3^Department of Radiotherapy, Habib Bourguiba Hospital, Sfax, Tunisia

## Abstract

The forkhead box (FOXA) family of transcription factors regulates gene expression and chromatin structure during tumorigenesis and embryonic development. Until now, the relationship between FOXA1 and the nasopharyngeal carcinoma (NPC) has not yet been reported. Therefore, our purpose is to analyze the expression of FOXA1 in 56 NPC patients compared to 10 normal nasopharyngeal mucosae and to correlate the expression with the clinicopathological features. Besides, we investigated the association between FOXA1 and LMP1 gene expression, as well as the EMT markers namely the E-cadherin and Twist1. Among 56 NPC tissues, 34 (60.7%) cases were positive for FOXA1. Furthermore, we noticed that FOXA1 expression correlated with TNM (*p* = 0.037), and age at diagnosis (*p* = 0.05). Moreover, positive expression of FOXA1 is likely to be associated with prolonged disease-free survival and overall survival rates. On the other hand, we observed a positive association between the expression of E-cadherin and FOXA1 (*p* = 0.0051) whereas Twist1 correlated negatively with FOXA1 (*p* = 0.004). Furthermore, knowing that LMP1 plays a key role in the pathogenesis of NPC, we explored the association of FOXA1 with the LMP1 gene expression in both NPC cell lines and tissues. We found that, in the C666-1 which displays low levels of LMP1, the expression of FOXA1 is high, and inversely in the C15 cell line that expresses a high level of LMP1, the level of FOXA1 is low. Besides, in accordance to our results, we found that in NPC tissues there is a negative association between LMP1 and FOXA1. In conclusion, our results suggest that the overexpression of FOXA1 is associated with a nonaggressive behavior and favorable prognosis in NPC patients. FOXA1 could contribute in the EMT process through key factors as E-cadherin, Twist1, and LMP1.

## 1. Introduction

The FOXA transcription factors promote gene expression and alter chromatin structure, thus allowing the binding of other factors that regulate transcription [1]. In mammals, this protein family comprises three members: FOXA1, FOXA2, and FOXA3 [2]. The expression and function of FOXA1 and FOXA2 overlap during the development of organs such as liver [3], lung [4], pancreas [5], and mammary gland [6]. The forkhead domain of the FOXA proteins is a DNA binding domain that binds to histones in the nucleosome of chromatin, causing its decompaction [7], allowing other transcription factors to bind to target genes. In human malignancies, the role of FOXA1 as pro- or antitumorigenic actor is not fully elucidated [8–10]. In breast cancer, overexpression of FOXA1 correlates with good prognosis in ER+ cases [11–18]. Whereas, in prostate cancer, overexpression of FOXA1 correlates with reduced survival rate [19]. In epithelial ovarian cancer, Wang et al. showed that FOXA1 may act as an oncogene, since silencing of FOXA1 in ovarian cancer cell lines decreased cell proliferation and increased cell apoptosis [20]. In both gastric and colorectal cancer, positive expression of FOXA1 correlated with adverse clinicopathological parameters and with poor 5-year overall survival [21, 22]. Moreover, in glioma cells and tissues, FOXA1 is overexpressed and induces the G1/S transition [23].

NPC is an epithelial tumor with a low incidence (1/100 000 individuals) in Europe and the USA; however, the endemic region was recorded in Southern China and Southeast Asia [24]. In North Africa, the incidence of NPC is intermediate (8/100 000 individuals) with two particular peaks, according to the age at diagnosis [25]. NPC is a particular entity of the head and neck cancers that is associated with Epstein-Barr virus (EBV) infection [26]. As a major oncoprotein of EBV, LMP1 (latent membrane protein 1), functions as a constitutively activated receptor [27, 28]. Indeed, LMP1 activity is similar to the receptor of the tumor necrosis factor (TNF) superfamily involved in several signaling pathways, as NF-*κ*B, p38/MAPK, and PI3K/Akt [28]. Consequently, LMP1 affects cell migration, proliferation, and apoptosis [28].

The purpose of this work is to study the expression levels of FOXA1 in primary NPC tissues and cell lines. Furthermore, the expression of FOXA1 was correlated with clinical parameters and EMT markers (E-cadherin and Twist1) expression in NPC tissues. Finally, knowing that LMP1 is a major actor in the development of NPC, we studied the association between LMP1 and FOXA1 expression.

## 2. Material and Methods

### 2.1. Patients and Cell Lines

This study enrolled 56 nasopharyngeal carcinoma (NPC) primary patients, from the Sfax region in the south of Tunisia. Frozen tissues were collected at the Department of Anatomo-Pathology from CHU Habib Bourguiba Sfax (Tunisia). In addition, 10 histological normal hyperplasia tissues were collected and used as controls. Prior to specimen collection, all individuals gave informed consent according to institutional guidelines. The clinicopathological characteristics of NPC patients included in this study are summarized in Table 1. The mean age is 47.98 years, (varying from 15 to 80 years). TNM and histological type were determined according to AJCC/UICC and WHO, respectively [29]. The overall survival (OS) and disease-free survival (DFS) data are available for only 30 among the 56 patients included in this study.

NPC cell lines C15, C666.1, and NP69 transfected or not with LMP1, derived from a primary NPC were provided by P. Busson, IGR, France. Tissues were frozen and stored at −80°C before any treatment.

### 2.2. RNA Extraction and cDNA Synthesis

TRIzol reagent (Invitrogen) was used to extract RNA from specimens according to the manufacturer's protocol; then, the RNA was quantified by NanoDropND-1000 (Thermo Scientific). RNA (300 ng) served as a template for cDNA synthesis using hexamer primers (50 pmol) and dNTP mix (10 nmol). After 10 min at 70°C, buffer, DTT (0.2 *μ*mol), and SuperScriptM II RTase (200 units) were added. After incubation for 12 min at 25°C followed by 50 min at 42°C, the reaction was stopped at 70°C for 15 min.

### 2.3. Real-Time and Semiquantitative RT-PCR

Quantitative RT-PCR (qRT-PCR) was performed using SYBR Green PCR Master mix RT-QPCR mix (Takara Bio). Primer sequences, amplicon size, and annealing T° are described in Table 2. GAPDH was used as an internal control, and the expression of FOXA1 and E-cadherin genes was normalized to the mean of all Cq values (CqCalib). Relative gene expression (NRQ) was calculated as follows: NRQ = 2ΔCqtarget/2ΔCqGAPDH, where ΔCqtarget = CqCalib target − Cqtarget and ΔCqGAPDH = CqCalibGAPDH − CqGAPDH, as described previously [30, 31].

For semiquantitative RT-PCR, aliquots of 100 ng of cDNA were used as a template with primers specific for Twist1 and LMP1 (Table 2) to generate 143 bp and 238 bp DNA fragments, respectively. GAPDH was used as an internal control.

### 2.4. Immunohistochemical Staining and Scoring

The samples obtained at surgery were routinely fixed in 10% neutral buffered formalin and embedded in paraffin. Before immunostaining, two pathologists (SC, TSB) reviewed haematoxylin and eosin-stained slides in order to select blocks representing tumor tissues. For each selected tumor, 4-*μ*m sections attached on poly-L-lysine-coated slides were fixed in acetone for 10 min and left to dry overnight at 37°C. Slides were deparaffinized in xylene followed by subsequent rehydration in graded ethanol. The sections were then pretreated with 3% hydrogen peroxide for 10 min to inactivate endogenous peroxides and washed in phosphate-buffered saline (PBS) solution. Heat-induced antigen retrieval was performed using epitope retrieval solution (Dako) at 95°C for 40 min. After heating, slides were allowed to cool down to room temperature and were briefly washed with PBS. A blocking solution (Dako) was used for 5 min to block the nonspecific binding of antibodies. Immunohistochemical staining was performed using the streptavidin–biotin peroxidase system (RE7280-K, Leica Biosystems). Tissue sections were incubated overnight with the primary antibody against FOXA1 (GTX34736, Gene Tex) for 30 min then with biotin-labeled secondary antibodies (Novolink Polymer, Leica Biosystems) and a streptavidin–peroxidase complex using diaminobenzidine as a chromogenic substrate (RE7280-K, Leica Biosystems). Immunostainings were scored on the basis of the percentage of positive tumor cells and the relative immunostaining intensity as described previously [28]. The FOXA1 immunostaining was interpreted as low (IS: ≤2), moderate (IS >2 and ≤4), and high (IS >4).

### 2.5. Statistical Analysis

GraphPad Prism 5.0 software was used to carry out statistical analysis and graphs. Data are expressed as the mean ± standard error of the mean (SEM). The nonparametric Mann–Whitney *U*-test was used for statistical evaluation of the differences between two independent groups. *p* < 0.05 was considered statistically significant. Univariate analysis for OS and DFS was performed by Kaplan-Meier analysis. Cox proportional hazard models were used to carry out multivariate survival using SPSS version 20.

## 3. Results

### 3.1. Expression of FOXA1 and Correlation with Clinicopathological Parameters in NPC

**Figure 1:**
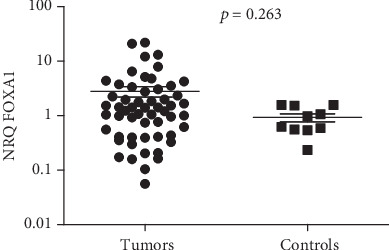
Expression levels of FOXA1 in NPC tissues (56 cases) and in normal nasopharyngeal mucosa (10 cases).

**Figure 2:**
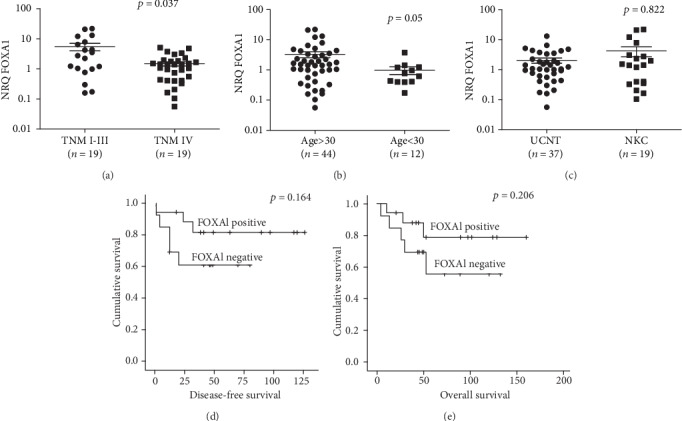
Association of the expression levels of FOXA1 with TNM (a), age (b), and histological type (c) in NPC. Correlations were tested using the nonparametric Mann–Whitney test. Kaplan-Meier plots showing the association of FOXA1 expression with the disease-free survival (d) and overall survival (e).

The expression level of FOXA1 was investigated in 56 NPC cases, and 10 normal nasopharyngeal mucosae were used as controls by RT-qPCR. The NRQ varied from 0.056 to 22 (mean = 2.793; 95%CI = 1.593 − 3.993) in NPC tissues, and the values ranged from 0.235 to 1.577 (mean = 0.927; 95%CI = 0.57 − 1.281). The level of FOXA1 was slightly higher in NPC tissues than in normal nasopharyngeal mucosa (*p* = 0.263, Figure 1). The expression of FOXA1 was considered as positive when the NRQ value is >1. Among 56 NPC tissues, 34 (60.7%) cases were positive for FOXA1. Significant associations between the expression of FOXA1 and TNM, and age at diagnosis were seen, but there is no association with the histological type (*p* = 0.037, *p* = 0.05, and *p* = 0.822, respectively, Figures 2(a), 2(b), and 2(c)). Interestingly, high FOXA1 levels were seen in tumors at TNM I-III, and in patients over 30 years.

Furthermore, Kaplan-Meier plots showed that patients with tumors positive for FOXA1 expression have a benefit in terms of DFS (*p* log − rank = 0.164) and OS (*p* log − rank = 0.206) (Figures 2(d) and 2(e)). Furthermore, Cox-regression analysis including the following parameters: FOXA1 expression, TNM-stage, pT-stage, lymph-node metastasis, and histological type showed that only FOXA1 expression is an independent factor for OS, while TNM stage, histological type, and FOXA1 expression are independent factors for DFS (Tables 3(a) and 3(b)).

**Figure 3:**
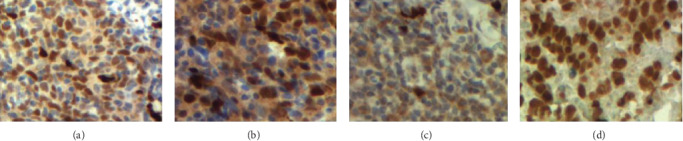
Immunohistochemical staining of FOXA1 in NPC. Representative images for negative (a), and positive (b) nuclear IHC staining of FOXA 1. Nontumoral nasopharyngeal mucosa (c), and prostate cancer (d) served as negative and positive controls, respectively.

On the other hand, we analyzed the expression of FOXA1 by IHC on 20 available NPC tissues. Representative examples of FOXA1 immunostaining in NPC are shown in Figure 3. Based on the immunostaining score, we found that the FOXA1 expression level was positive (high or moderate) in 55%, and low (negative or weak) in 45% of tumor tissues (Figures 3(a)–3(d)).

### 3.2. Expression of FOXA1 and EMT Markers in NPC

**Figure 4:**
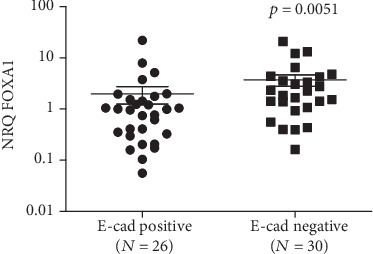
Association of FOXA1 expression level with E-cadherin in NPC tissues. Correlations were tested using the nonparametric Mann–Whitney test.

The expression of two EMT markers, namely the E-cadherin and Twist1, was analyzed and associated with FOXA1 in NPC patients. The NRQ values for E-cadherin varied from 0.05 to 19.45 (mean = 2.65, 95%CI = 1.502 − 3.8), and the expression was considered as positive if the NRQ value is >1. Among 56 cases, 26 were E-cadherin positive and associated with FOXA1 (*p* = 0.0051, Figure 4).

**Figure 5:**
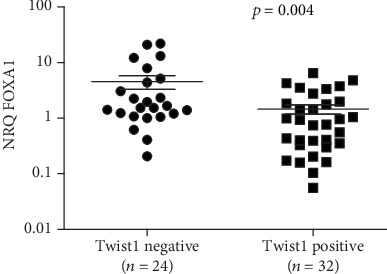
Association of FOXA1 expression levels with Twist1 in NPC tissues. Correlations were tested using the nonparametric Mann–Whitney test.

Furthermore, to study the relationship between FOXA1 and Twist1, we performed semiquantitative RT-PCR. Twist1 mRNA was detected in 32 cases, whereas 24 samples were negative. Using the nonparametric Mann–Whitney test, we found a negative association between the average expression level of Twist1 and FOXA1 (*p* = 0.004, Figure 5).

### 3.3. Relationship between LMP1 and FOXA1 in NPC

**Figure 6:**
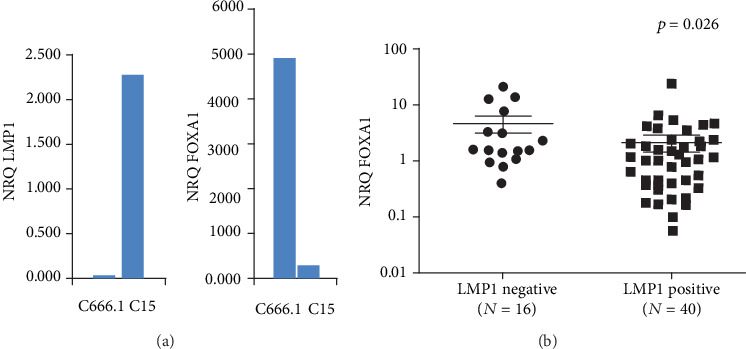
The expression of EBV's oncoprotein: LMP1 compared with FOXA1 expression in NPC derived cell lines (a) and its association with FOXA1(b) in NPC tissues.

LMP1 is the major EBV oncoprotein involved in the development of NPC. Therefore, we examined the association between LMP1 and FOXA1 in NPC cell lines and tissues. The expression level of LMP1 is drastically different in the two NPC cell lines C666-1 and C15. Indeed, LMP1 is overexpressed in C15 but very low in C666-1 (Figure 6(a)). Interestingly, we found that FOXA1 expression inversely correlated with LMP1 in NPC cell lines (Figure 6(a)). In NPC samples, LMP1 expression was detected in 40 among 56 cases, and positive LMP1 tissues showed low levels of FOXA1 (*p* = 0.026, Figure 6(b)).

### 3.4. Effect of LMP1 on FOXA1 and EMT Markers in NPC Cell Line

**Figure 7:**
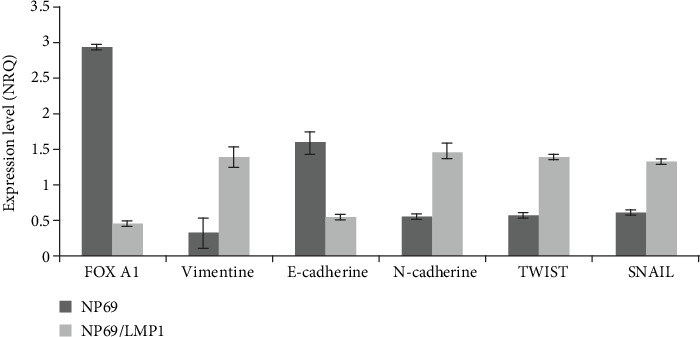
Gene expression analysis by RT q-PCR of FOXA1 and EMT markers in NP69 and NP69/LMP1 cell lines.

The expression of FOXA1 and EMT markers was analyzed in NP69, a NPC-derived cell line transfected or not with the EBV oncoprotein LMP1. As demonstrated in our study on NPC patients, FOXA1 is overexpressed, while Twist-1 is downregulated in the absence of LMP1 expression (NP69). Regarding mesenchymal markers (vimentin and N-cadherin), we noticed gene overexpression in the presence of LMP1 (NP69/LMP1). However, the expression of the epithelial marker (E-cadherin) decreased in NP69/LMP1 cell line (Figure 7). Furthermore, in NP69/LMP1, the expression of Snail increased compared to NP69 (Figure 7).

## 4. Discussion

NPC is a particular type of head and neck cancer with a high metastatic potential [26]. Microarrays and WES have facilitated the identification of several candidate biomarkers such as FJX, WNT5A, CLDN1, FGFR3, FZD6, and SOX4. Among these genes, it was reported that the four-jointed box 1 (FJX1) is overexpressed in NPC tissues and promotes cell proliferation through regulating Cycline D1 and E1 [32]. Furthermore, several key signaling pathways such as Notch, NF-*κ*B, and PI3K/Akt were frequently altered during NPC development and progression [26, 33, 34]. Tang et al. demonstrated that the transcription factor SOX2 upregulated expression of PIK3CA through interacting with KLF4 to activate downstream PI3K/AKT signaling pathways that enhance tumorigenesis [35].

FOXA1 has been linked to various types of tumors [18–23, 36]; however, its involvement in NPC has not yet been explored. In this study, FOXA1 expression was analyzed in NPC patients, and we found that among 56 NPC tissues, 34 (60.7%) cases were positive for FOXA1. In addition, IHC showed that 55% of NPC cases displayed positive FOXA1 expression confirming our data on mRNA levels.

FOXA1 is significantly overexpressed in TNM stages (I-III) suggesting that FOXA1 decreases tumor progression in NPC. On another hand, it is well established that the juvenile form of NPC in North African patients is more aggressive with frequent relapse than in adult patients [25]. Interestingly, our results showed that FOXA1 is downexpressed in young patients confirming that the low level of FOXA1 is related to aggressiveness in NPC. Furthermore, patients with tumors positive for FOXA1 expression have a prolonged overall survival and disease-free survival rates. This result is in concordance with previous works reporting that FOXA1 expression correlated with better prognosis in breast cancer [17–19]. Recently, Peng et al. demonstrated that FOXA1expression induced remarkable transcriptomic changes in miRNAs as well as mRNAs levels in the NPC HK1 cell line [37]. They showed that FOXA1 repressed two oncogenic miRNAs miR-100-5p and miR-125b-5p, to upregulate their corresponding target, RASGRP3 or FOXN3 genes that contributed to the inhibition of malignant behaviors of NPC cells [37].

In epithelial ovarian cancer, Wang et al. showed that overexpression of FOXA1 is associated with an increased WHO grade, poor differentiation, and reduced overall survival time [20]. In gastric cancer, FOXA1 acts as an oncogene by inhibiting apoptosis and activating cell proliferation rate [21]. Moreover, in glioma cells and tissues, FOXA1 is overexpressed and induces the G1/S transition [23]. Altogether, these data indicate that FOXA1 can promote tumor progression or suppress tumor development depending on tumor type, which requires further studies to elucidate the function of FOXA1in tumor progression.

In a recent work, Li et al. performed microarray analysis on control versus FOXA1 expressing NPC cells. They found 298 differentially regulated genes and pathway analysis of the FOXA1-upregulated genes revealed that the TGF-*β* signaling pathway is perturbed in NPC [38]. They showed that FOXA1 acts as a tumor suppressor in NPC development through the control of the TGF-*β* stimulated transcription program. In the absence of FOXA1, TGF-*β* activates oncogenic target gene expression including PTHLH, L1CAM, PHLDA1, HMGA2, and vimentin. In contrast, the presence of FOXA1 enhanced tumor suppressor gene expression, including that of PMEPA1, LMO7, and BAMBI [38].

Several studies demonstrated that FOXA1 contributes to the epithelial-mesenchymal transition (EMT) mainly through regulating E-cadherin expression [39, 40]. Our data showed that the expression of FOXA1 correlated positively with E-cadherin which is in line with previous reports [39, 40]. Anzai et al. reported that FOXA1 promotes E-cadherin expression at the protein level by suppressing Slug expression in MCF7 cells [41]. Similarly, in gastric cancer cells, FOXA1 regulates the EMT in cancer cells, by inducing the E-cadherin expression and decreasing the vimentin protein level [42].

Among the EMT-transcription factors, Twist1 induces the EMT in cancer cells resulting in tumor progression and invasion [43]. Mechanistically, Twist1 recruits the nucleosome remodeling deacetylase (NuRD) complex which in turn represses the E-cadherin expression and induces EMT in breast cancer [44]. Our data showed that tumors which are negative for Twist1, significantly overexpressed FOXA1 in NPC patients confirming that Twist1 regulates negatively FOXA1 which in turn inhibits the expression of E-cadherin in NPC. NPC is an EBV-associated tumor which often expresses the latent membrane protein 1 (LMP1) [27, 28]. This viral oncoprotein can activate the EMT cascade by inducing Twist1 via the nuclear factor-*κ*b (NF*κ*b) signalization pathway in nasopharyngeal epithelial cells and NPC tissues [44]. Based on the well-established relationship between LMP1 and Twist1 in NPC, we analyzed whether FOXA1 could be associated with LMP1 expression. In NPC cell lines displaying a low or high level of LMP1 (C666-1 and C15, respectively), we found that FOXA1 is inversely associated with LMP1suggesting that the oncoprotein probably regulates the expression of FOXA1 via Twist1. Further studies should be done to better elucidate the regulation between LMP1-Twist-FOXA1 in NPC. Although, Horikawa et al. [45] have clearly demonstrated the role of LMP1 in the induction of Twist via NF-*κ*B pathway; the underlying mechanisms between LMP1 and FOXA1 are still unclear. Our data on NPC cell lines were confirmed by NPC tissues where a significant inverse correlation was seen between LMP1 and FOXA1.

Furthermore, we investigated the expression of FOXA1 and EMT markers in NP69, a NPC-derived cell line transfected or not with the EBV oncoprotein LMP1. We confirmed our data found with NPC patients, showing that FOXA1 is overexpressed and Twist-1 is downregulated in the absence of LMP1 expression. In addition, we noticed overexpression of mesenchymal markers (vimentin and N-cadherin), in the presence of LMP1 (NP69/LMP1); however, the expression of the epithelial marker (E-cadherin) decreased in NP69/LMP1 cell line. Horikawa et al. reported that LMP1 induces Snail and the epithelial to mesenchymal transition in metastatic NPC tissues [46] which is in agreement with our data observed on NP69 and NP69/LMP1 cell lines.

In summary, FOXA1 is overexpressed in NPC and associated with EMT markers, namely E-cadherin and Twist. Besides, given the key role of LMP1 in NPC, we showed that this oncoprotein could regulate FOXA1 expression probably via Twist1 that in turn promotes the EMT process in NPC. However, further functional studies are needed to explore the molecular mechanisms behind the association of FOXA1 and LMP1 in NPC.

## 5. Conclusion

Our results suggest that the overexpression of FOXA1 is associated with a nonaggressive behavior and favorable prognosis in NPC patients. FOXA1 could contribute in the EMT process through key factors such as E-cadherin, Twist1, and LMP1.

## Figures and Tables

**Table 1:** Clinicopathological characteristics of NPC patients.

Variables	*N*
Age	
≤30	12
>30	44
Gender	
Male	17
Female	10
TNM stage	
I	2
II	8
III	9
IV	31
Histological type	
NKC	19
UCNT	37
Tumor stage	
T1-T2	20
T3-T4	30
Lymph node metastasis	
N0-N1	11
N2-N3	39
Distant metastasis	
M0	42
M1	8

**Table 2:** Primer sequences for RT-PCR and RT-qPCR.

Gene		Primer sequences (5′-3′)	Product size (bp)	Annealing T (°C)
FOXA1	Forward	CGCTTCGCACAGGGCTGGAT	143	62
	Reverse	TGCTGACCGGGACGGAGG AG		
E-cadherin	Forward	CGGGAATGCAGTTGAGGATC	199	60
	Reverse	AGGTGGTGTAAGCGATGGC		
N-cadherin	Forward	ATTGGACCATCACTCGGCTTA	158	58
	Reverse	CACACTGGCAAACCTTCACG		
LMP1	Forward	AGCCCTCCTTGTCCTCTATTCCTT	253	60
	Reverse	ACCAAGTCGCCAGAGAATCTCC		
TWIST	Forward	CAAGCTGCAGCTATGTGGC	168	57
	Reverse	TGTCCATTTTCTCCTTCTCTGG		
Vimentin	Forward	CCCTCACCTGTGAAGTGGAT	271	60
	Reverse	TCCAGCAGCTTCCTGTAGGT		
Snail	Forward	GATGCCGCGCTCCTTCC	265	61
	Reverse	GGGGACTCACTCGCCCC		
GAPDH	Forward	GCTCTCTGCTCCTCCTGTTC	122	60
	Reverse	CGCCCAATACGACCAAATCC		

**(a):** 

Covariates	*P* value	HR^a^	95% CI^b^	
			Lower	Upper
FOXA1 expression	0.051	0.214	0.046	1.004
pT-stage	0.645	1.211	0.536	2.736
Histological type	0.237	0.342	0.058	2.022
N-stage	0.475	1.419	1.294	8.86
TNM-stage	0.298	2.812	0.401	19.741

HR^a^: hazard ratio; 95% CI^b^: confidence interval.

**(b):** 

Covariates	*P* value	HR^a^	95% CI^b^	
			Lower	Upper
FOXA1 expression	0.031	0.093	0.011	0.809
pT-stage	0.244	0.543	0.195	1.515
Histological type	0.523	1.88	0.271	13.044
N-stage	0.05	0.072	0.005	0.996
TNM-stage	0.014	13.62	2.682	6.924

HR^a^: hazard ratio; 95% CI^b^: confidence interval.

## Data Availability

The data sets used and/or analyzed during the current study are available from the corresponding author on reasonable request.
